# Sub-inhibitory Effects of Antimicrobial Peptides

**DOI:** 10.3389/fmicb.2019.01160

**Published:** 2019-05-24

**Authors:** Alexey S. Vasilchenko, Eugene A. Rogozhin

**Affiliations:** ^1^Institute of Environmental and Agricultural Biology (X-BIO), Tyumen State University, Tyumen, Russia; ^2^Shemyakin–Ovchinnikov Institute of Bioorganic Chemistry, Russian Academy of Sciences, Moscow, Russia; ^3^Gause Institute of New Antibiotics, Moscow, Russia

**Keywords:** antimicrobial peptides, AMP, sub-inhibitory effects, virulence, factor of pathogenicity

## Abstract

Antimicrobials, and particularly antimicrobial peptides (AMPs), have been thoroughly studied due to their therapeutic potential. The research on their exact mode of action on bacterial cells, especially at under sublethal concentrations, has resulted in a better understanding of the unpredictable nature of bacterial behavior under stress conditions. In this review, we were aiming to gather the wide yet still under-investigated knowledge about various AMPs and their subinhibition effects on cellular and molecular levels. We describe how AMP action is non-linear and unpredictable, also showing that exposure to AMP can lead to antimicrobial resistance via triggering various regulatory systems. Being one of the most known types of antimicrobials, bacteriocins have dual action and can also be utilized by microorganisms as signaling molecules at naturally achievable sub-inhibitory concentrations. The unpredictable nature of AMP action and the pathogenic response triggered by them remains an area of knowledge that requires further investigation.

## Introduction

Antimicrobial peptides (AMPs) are protective molecules of innate immunity in living organisms (Zasloff, 2002).

In general definition, antimicrobial peptides are a diverse group of naturally derived or synthetically obtained molecules, which have antimicrobial properties because of their specific physical properties (antivirus and/or antitumor properties, in several cases). Attempts to classify antimicrobial peptides interfere with the structural diversity of existing substances. In a general, there are two ways in which peptides are synthesized; this fact underlies their structural and functional diversity. Natural-derived AMPs can be formed by ribosomal synthesis and can be produced from non-ribosomal peptide synthesis. Ribosomally synthesized peptides are produced by almost all organisms, their classification is based on the secondary structure formed in aqueous solutions. Thus, distinguish α-helical, β-sheet, peptides with extended/random-coil structure (Hancock and Chapple, 1999; Bahar and Ren, 2013; Mahlapuu et al., 2016).

In turn, the greatest diversity is inherent in microbial antimicrobial peptides, since microorganisms are capable not only of non-ribosomally synthesis (Hancock and Chapple, 1999), but also of post-translational/co-translational modifications (Arnison et al., 2013). Extensive post-translational modifications give peptides additional properties, for example, better recognition of targets and increased stability, which expands their functionality as compared to ribosomally synthesized peptides of animals (Arnison et al., 2013). These peptides have been classified within the bacteriocins, the most recent classification of which is given in review (Acedo et al., 2018).

As of now, the nature of antimicrobial peptides has been thoroughly investigated. All data accumulated to date can be summarized in simple statistics. For instance, upon a query, an antimicrobial peptide database (October 2018) returns extracted data on three thousand peptides with annotated structures (Wang et al., 2016).

In addition, the number of articles dedicated to the study of antimicrobial peptides exceeds 350,000^1^. Such a heightened interest in this topic does not seem unreasonable, since antimicrobial peptides remain an attractive alternative to conventional antibiotics. AMPs have a unique ability to overcoming pathogenic virulence and defense, primarily by targeting highly conserved structures of the microbial cell (Brogden, 2005; Omardien et al., 2016). Due to the unique properties of AMPs, they can and should be used for the benefit of humanity in the face of the antibiotic resistance catastrophe (Ventola, 2015). Existing efforts of scientific research are directed toward searching for more effective bactericides and studying of their mode of action (Cytryńska and Zdybicka-Barabas, 2015). Even though such investigations are necessary, there are some aspects of this problem that are poorly addressed by research. This includes the under-investigated effects of sub-inhibitory concentrations (sub-MIC) of AMPs on the physiology of the bacterial cells. Often, produced peptides dilute in the environment medium. Thus, it appears that the peptide concentration necessary for bactericidal of fungicidal effect is not always achievable in natural conditions.

Regarding conventional antibiotics, their effects at sub-inhibitory concentrations have been studied for a substantially long period of time (Lorian, 1975; Andersson and Hughes, 2014). It has been shown that sub-inhibitory concentrations of antibiotics can trigger unexpected reactions from the bacterial population. For example, fluoroquinolones can stimulate bacterial adaptation to different stresses, including effects of antibiotics (López and Blázquez, 2009).

By the way the AMP’s action on eukaryotic cells also have concentration dependent features (Baindara et al., 2017).

Generally, the antimicrobial action of peptides is exhibited via compromising the integrity of the microbial cell’s barrier structures. However, other intracellular targets for peptides are known (Hale and Hancock, 2007), which leads to the conclusion about peptide’s multifunctional nature (Le et al., 2017). In this review, we are summarizing the currently available data on the sub-inhibitory concentrations effects (sub-MIC effects) of antimicrobial peptides on bacteria. Our main interest is directed toward peptides’ ability to trigger various effects on subcellular (expression of virulence genes) and cellular (phenotypic manifestation of the response) levels. It is important to note that the response of a bacterial population to AMP’s treatment can be both positive and negative for humans. Positive effects include changes in the morphofunctional properties of bacteria that, lead to a decrease in their pathogenicity. Negative effects are comprised of increased bacterial aggression after being exposed to antimicrobial peptides.

The remaining questions are as follows:

1. What are the triggering mechanisms behind sub-MIC effects?
2. Is it possible to predict the nature of the bacterial response to sub-MIC action of an AMPs?
3. How exactly does AMP structure determine its sub-MIC action?

Given the therapeutic potential of antimicrobial peptides in addition to the known data on the sub-MIC effects of conventional antibiotics, this review aims to encourage the investigation on the non-killing effects of antimicrobial peptides.

## Sub-Inhibition Concentration Effects of AMPs at Subcellular Level

### The Molecular Mechanisms of Peptide Reception and Response to Sub-inhibitory Action

Antimicrobial peptides have physical and chemical properties necessary to be able to interact with bacterial membranes (Datta et al., 2015). Interaction of cationic peptides is promoted through electrostatic interaction, while interaction of anionic peptides is driven by hydrophobicity (Phoenix et al., 2013; Travkova et al., 2017). Membrane damage is the main cause of cell death, since it disrupts the work of many subsystems, associated with the membrane’s integrity. If membrane damage is not fatal, the cell is able to respond to external stress.

Bacterial genomic machinery responds with the expression of various genes within several minutes after the moment of exposure to stress factors. One of the first works on sub-MIC effects of AMPs was dedicated to cecropin A and *E. coli* cells (Table 1). It was found that cecropin A caused a significant change in the transcript levels for 26 bacterial genes (Hong et al., 2003); the sub-MIC of colistin altered expression of 30 genes of *P. aeruginosa* (Cummins et al., 2009); LL-37 affected expression of several 100 genes of *P. aeruginosa* (Overhage et al., 2008).

**Table 1:** The physico-chemical properties of antimicrobial peptides described in the review.

Peptide	Mol. weight, Da^1^	Type of structure	Charge^2^	μHrel^2^	GRAVY^1^	The sources of the structural data
LL-37	4490.6	Alpha-helix conformation	+6	0.499	−0.72	www.anaspec.com
Cecropin A	4004.82	Alpha-helix conformation	+6	0.202	−0.07	www.anaspec.com
Indolicidin	1700	Random coil	+4	0.190	−1.07	www.anaspec.com
Fallaxin analog FL9	2717	Alpha-helix	+2	0.275	0.51	Nielsen et al., 2007
C18G	2043	Alpha-helix	+7	0.604	−0.19	Kohn et al., 2018
α-defensin HNP-1	3448	β-strand, β-turn	+3	0.028	0.30	www.anaspec.com
β-defensin hBD-1	3934	Alpha-helix and triple-stranded antiparallel β-sheet	+4	0.348	−0.27	www.anaspec.com
β-defensin hBD-2	3885.9	Alpha-helix and triple-stranded antiparallel β-sheet	+6	0.246	−0.10	http://bpsbioscience.com/bd-2-90107-b
Bovicin HC5	3525		+2	0.163	0.28	http://bactibase.hammamilab.org
Subtilosin A	3425	Cysteine sulfur to α-carbon bridges	−2.2	0.08	0.69	Acedo et al., 2018, http://bactibase.hammamilab.org
Plantaricin A	2683	Alpha-helix conformation	+5	0.321	−0.24	http://bactibase.hammamilab.org
Subtilin	3465	Fivefold-stranded antiparallel β-sheet and alpha-helices	+2	0.151	0.19	http://bactibase.hammamilab.org
Nisin Z	3475	Alpha-helices and β-turn	+3	0.084	0.41	http://bactibase.hammamilab.org
Polymyxin B	1203.50	Cyclic	+5	ND	ND	Fernández et al., 2012
Colistin	1156.0	Cyclic	+5	ND	ND	Fernández et al., 2012
Hemoglobin-derived Hbg-1	2495	Random coil	+1	0.053	−0.56	Merriman et al., 2014
Hemoglobin-derived Hbg-2	2495	Random coil	+1	0.220	−0.56	Merriman et al., 2014
Dipeptides cyclo(L-Phe-L-Pro)	245.35	ND	ND	ND	ND	Li et al., 2011

*^1^The properties were calculated using the web-tool, which is available at https://www.thermofisher.com/ru/ru/home/life-science/protein-biology/peptides-proteins/custom-peptide-synthesis-services/peptide-analyzing-tool.html. The grand average of hydropathicity (GRAVY) of a peptide is the sum of the hydropathy values of all the amino acids divided by the number of residues in the peptide or protein sequence. ^2^The properties were calculated using the web-tool, which is available at http://heliquest.ipmc.cnrs.fr/cgi-bin/ComputParams.py. The relative hydrophobic moment (μHrel) is the hydrophobic moment of a peptide relative to that of a perfectly amphipathic peptide.*

Thus, antimicrobial peptides in the non-killing concentration has a strong restructuring effect on of a genome’s functionality.

### Can the Direct Peptide-DNA Interaction Affect Bacterial Transcriptome?

What is the mechanism of signal reception and transmission? It may be a direct interaction of the peptide molecule with bacterial DNA. It is known that many AMPs have a dual mode of action (Table 2). At high peptide concentrations they cause damage to cell membranes, eventually breaking it down, but at lower concentrations, peptides translocate to the cytoplasm and electrostatically interact with DNA or ribosome (Gottschalk et al., 2015; Polikanov et al., 2018). For example, a number of synthetic peptides can interact with DNA and induce a SOS-response. During this process, peptide’s action increases the expression of the α-haemolysin (Gottschalk et al., 2015). A similar effect was shown for indolicidin, which disturbed a membrane at MIC and induced the SOS-response at sub-MIC (Vasilchenko et al., 2017). The direct mutagenic effect of the cationic peptide is known (Limoli et al., 2014). However, it should be noted that mutagenesis and SOS-response are observed only at concentrations close to MIC, whereas a change in the transcriptome is usually observed at doses that are many times smaller (Farris et al., 2010).

**Table 2:** The mode of action and sub-inhibitory effects of peptides described in the review.

Peptide	The cell’s targets	Negative sub-MIC effects^∗^	Positive sub-MIC effects^∗^	References
LL-37	Membranes permeabilization; direct DNA binding	Promote mucoidy phenotype in Gr-bacteria; overproduction of virulence factors; promote resistance to antimicrobials	Inhibites biofilm formation *P. aeruginosa* and *S. aureus*	Fernández et al., 2010; Dean et al., 2011; de la Fuente-Núñez et al., 2012; Strempel et al., 2013; Limoli et al., 2014
Cecropin A	Membranes permeabilization	Unknown	Unknown	Silvestro et al., 2000; Hong et al., 2003; Rangarajan et al., 2013
Indolicidin	Membranes permeabilization; direct DNA binding	Promote resistance to antimicrobials	Prevent biofilm development of MRSA *S. aureus*	Fernández et al., 2010, 2012; Mataraci and Dosler, 2012
Fallaxin analog FL9	Membranes permeabilization; direct DNA binding	Increase production of α-haemolysin	Unknown	Gottschalk et al., 2015
C18G	Membranes permeabilization	Increased expression of the virulence factor of *S. typhimurium*	Unknown	Yu and Guo, 2011
α-defensin HNP-1	Membranes permeabilization; lipid II binding; target the ExPortal of *S. pyogenes*	Unknown	inhibition of secretion of SpeB cysteine protease and the streptolysin O	Vega and Caparon, 2012
β-defensin hBD-2	Membranes permeabilization	Unknown	Regulatory of gut homeostasis	Marzani et al., 2012; Dicks et al., 2018
Bovicin HC5	Membranes permeabilization	Unknown	Prevents biofilm formation of *S. aureus*	Mantovani et al., 2002; Pimentel-Filho Nde et al., 2014
Subtilosin	Membranes permeabilization	Unknown	Prevents biofilm formation of Gram-negative bacteria	Algburi et al., 2017
Plantaricin A	Membranes permeabilization at high (*in vitro*) concentration and pheromone at low (in natural) concentration	Unknown	Involved in the formation of a sustainable animal microbiome	Anderssen et al., 1998; Hauge et al., 1998; Kristiansen et al., 2005; Sturme et al., 2007; Calasso et al., 2013
Nisin	Membranes permeabilization; inhibites peptidoglycan sintesis; pheromone	Unknown	Inhibites bacterial biofilm formation	Mahdavi et al., 2007; Shin et al., 2015
Polymyxin B	Membranes permeabilization	Promote resistance to antimicrobials	Inhibites of secretion of SpeB cysteine protease and the streptolysin O	Fernández et al., 2012; Vega and Caparon, 2012
Colistin	Membranes permeabilization	Resistance; promote biofilm formation; pyocyanin production		Cummins et al., 2009; Fernández et al., 2012
Hemoglobin-derived peptides (Hbg-1, 2 and other)	Membranes permeabilization	Promote *S. aureus* surface colonization	Inhibites production of TSS toxin-1, enterotoxin C, α, δ hemolysin of *S. aureus*	Schlievert et al., 2007; Pynnonen et al., 2011
Dipeptides cyclo(L-Phe-L-Pro)	Unknown	Unknown	Inhibites production of TSS toxin-1	Li et al., 2011

*^∗^Is meant the reactions of the bacterial population, which has a final positive or negative effect on macroorganism (animal, plant, etc.).*

Thus, changes in gene expression caused by the DNA-peptide interaction should be considered exceptional and not as a general rule.

Recently a novel approach for precisely prevention of pathogenicity of Gram-negative bacteria was described, which is based on blocking a specific gene transcription by cationic peptide. The authors designed and synthesized cationic hydrocarbon stapled alpha-helical peptides based on a DNA-interacting a helix of σ54. The treatment of bacteria with synthesized peptides blocked the interaction between endogenous σ 54 and its target DNA sequence (Payne et al., 2018).

Thus, deciphering the molecular mechanisms of interaction of peptides with intracellular targets is a bridge between the fundamental knowledge and the practical use of the knowledge gained.

### Peptide Sensing?

In addition to nucleic acids, there are other intracellular targets for antimicrobial peptides. In particular, the bacterial cell envelope contains a variety of sensory regulatory systems, which sense environmental signals and regulate a genes expression accordingly.

Two-component systems (TCS) are widely distributed among bacteria and are diverse in structure and function. The presence of about one hundred thousand identified and classified TCS allows bacterial cells to recognize many different stressors and respond to them (Tiwari et al., 2017). In general, a TCS is comprised of a sensor protein (histidine kinase) and its corresponding response regulator. The sensor kinase attaches to the bacterial cytoplasmic membrane that has a sensing domain on its extracellular side.

**FIGURE 1:**
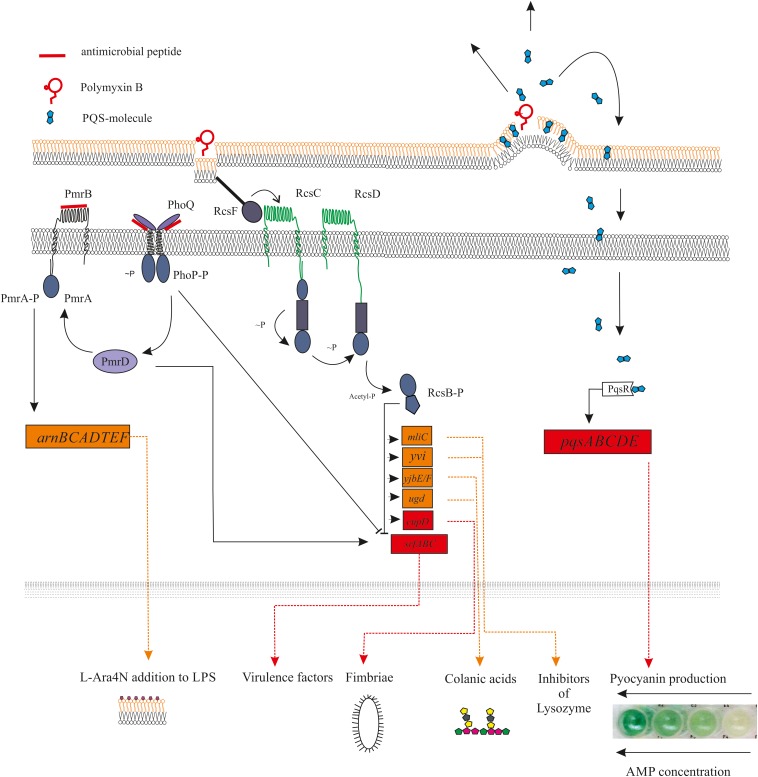
Diagram showing the main receptors of antimicrobial peptides and the relationship between them. The mechanisms of signal transmission from the activated receptor to the corresponding genes are shown. Antimicrobial peptides is capable to direct (PhoQ, PmrAB, and other) or indirect (RcsF) activation of the histidine kinase sensors, which led to regulation of activity of appropriated genes. At the same time, the expression of some genes can simultaneously be under the positive and/or negative regulation of different TCS. For example, the effect of polymyxin B on the bacterial outer membrane can activates the RcsF sensors, what leads to inhibition of expression of virulence genes in *srfABC* operon. Interestingly, the same operon is activated by another TSC PmrAB, for which “Peptide Sensing” was revealed (for example, for LL-37).

Antimicrobial peptides can have an effect on bacterial genomes both indirectly and directly. Indirect action occurs in response to a violation of the structural integrity of cell barriers (Table 2). For example, Rcs regulon controls the expression of many specific virulence factors in bacteria belonging to the Enterobacteriaceae family. According to a model proposed by Farris et al. (2010), the sensory molecule RcsF is anchored to the outer membrane, sequestered from its signaling partners in the “off state.” During the cellular envelope disorganization, conformational or spatial change promote direct non-covalent interaction of RcsF with periplasmic domains of signaling constituents, leading to Rcs activation. A more detailed molecular mechanism is described in the review (Guo and Sun, 2017; Figure 1).

Interaction of antimicrobial peptides with bacterial membranes in some cases led to an indirect activation of several genes regulated through “Quorum Sensing” (QS). It is known that some hydrophobic QS-autoinducers such as PQS are trafficked between cells via membrane vesicles (Mashburn-Warren et al., 2008). In this case, the peptide’s membrane-permeabilizing action releases accumulated PQS molecules, which can triggers the expression of the virulence genes associated with quorum sensing (Cummins et al., 2009; Figure 1).

Another example of TCS being indirectly activated by AMPs is the PhoQP two-component system, which controls the development of resistance to AMPs. The periplasmic domain of the PhoQ sensor is in conjunction with Mg^2+^ cations. Reducing the available amount of magnesium leads to electrostatic repulsion between PhoQ and the inner membrane domain (Cho et al., 2006). The resistance of *Salmonella* to polymyxin B is formed through this mechanism, since this AMP is able to displace Mg^2+^ cations from their binding site in the PhoQ sensor (Santos et al., 2017; Figure 1).

The majority of antimicrobial peptides have cationic properties that allow them to interact directly with the extracellular loop of sensors activating them (Li et al., 2007b; Gryllos et al., 2008). The possibility of such direct interaction was convincingly demonstrated in the study examining the ability of the LL-37 to activate the expression of streptococcal virulence factors, which are under control of the CsRS (CovRS) two-component system (Gryllos et al., 2008). Streptococci have cell surface-associated histidine kinases CsrS that can directly sense peptide molecules (Tran-Winkler et al., 2011). It turned out that a 10-amino acid residue fragment of the LL-37 did not exhibit any antimicrobial activity, but it determined the direct interaction of the LL-37 molecule with the sensory part of CsrS, according to the principle of ligand-receptor interaction (Velarde et al., 2014). Presumably, such interactions are determined by electrostatic forces, since the sensor domain of a two-component system has periplasmic loops which are usually negatively charged (Fernández et al., 2010).

Thus, there is strong evidence for the fact that bacteria have some kind of “Peptide Sensing.” It is only left to find out how sensitive is the “Peptide Sensing.” Does the “Peptide Sensing” recognize the specific structure of a peptide or does it responds to peptides as stress agents in the whole? These questions are not easy to answer, and more research is still needed. However, it is already clear that bacteria have sensory systems and mechanisms, which respond specifically to positively charged amphiphilic molecules with a certain amino acid composition.

### Qualitative and Quantitative Response of Sensory Regulatory Systems on Antimicrobial Peptides

Sensory systems can be categorized depending on their ability to recognize peptide structural features. The sensory systems are triggered by molecules with cationic and amphiphilic properties and constitute the first level of defense, since the primary result of their activation is the development of resistance to AMPs. For example, Rcs phosphorelay systems are activated through outer membrane disturbance only by hydrophobic substances like most antimicrobial peptides (Farris et al., 2010). In turn, the sensory part of the *aps* three-component system of staphylococci can recognize a variety of cationic, but not anionic AMPs (Li et al., 2007a).

The second level consists of sensory systems, which are possibly activated with a wide range of different peptides. Their quantitative properties are crucial. For example, the PhoQP TCS is activated by peptides with various structures, but the more charged and hydrophobic the peptide is, the greater activation is achieved by the exposure to it (Shprung et al., 2012). Thus, it was shown that LL-37, but not polymyxin B, activates the expression of virulent genes, which are under the control of PhoQP/PmrAB (Shprung et al., 2012). The used peptide’s sub-MIC concentrations are also important for the final result. For example, sub-MIC effect of LL-37 on *Pseudomonas aeruginosa* PAO1 at 4 μg/mL was down-regulation of QS-gene (*pqsE*) and other (production of rhamnosyltransferase, phenazine, etc.) (Overhage et al., 2008), but increase its expression at 20 μg/mL (Strempel et al., 2013).

It would be an interesting attempt to circumvent the undesirable sub-inhibitory effects by tuning of physic-chemical properties of designed synthetic peptides. Unfortunately, today there is no complete understanding to predict which of TCS will be activated. Various TCS have a different susceptibility to AMPs. Thus, using a bioluminescent reporter strain, it was shown that ParRS TSC was activated after being treated with colistin/polymyxin B and indolicidin, while other cationic peptides (including LL-37) did not activate it (Fernández et al., 2012). Additional experiments with 19 peptides, different in charge and hydrophobicity, did not reveal a clear correlation between peptides’ properties and their activation ability (Fernández et al., 2012). New targeted researches aimed to study the sub-inhibitory effects of AMPs in the structure-function aspect, with appropriate mathematical processing, would allow answering many questions.

Thus, these facts allow us to conclude that different sensory systems have different levels of sensitivity and the ability to recognize specific stressors. Ultimately, this determines the various responses of bacterial cells to different AMPs. However, it can be assumed that the main reaction of bacterial genome and its metabolic apparatus is developing resistance, while all other effects may be secondary. Probably, in stress conditions, this is the most adequate response of bacteria to the antimicrobial action of peptides, which, however, can be followed by others.

### Bacterial Defense Network Is Activated by AMPs

Numerous different genes that are directed toward following a forming network and regulate a comprehensive strategy of protection and response to external influences are under the control of one master regulator. The GraSR TCS of *S. aureus*, which are involved in AMPs resistance, and are indirectly associated with pathogenesis, control pathways through connections with Agr signal transduction network (Kraus et al., 2008; Falord et al., 2011). Bacterial Rcs phosphorelay is a well-known signaling system that regulates virulence and persistence of Enterobacteriaceae (Erickson and Detweiler, 2006). The Rcs, simultaneously with PhoQP and PmrAB TCS, is involved in regulation of several genes, whose expression maintained integrated resistance of bacteria to polymyxin B (Llobet et al., 2011; Figure 1).

There is a large number of similar examples, which shows a close interweaving of different ways of signal transmission and responding. Often, stress activates a variety of regulatory systems that overlap closely. Thus, while being surrounded by antimicrobial peptides, bacterial cells experience stress, the first response to which will be self-protection.

Concerning the peptides themselves, there is no doubt that their exclusive physicochemical properties are important. However, a more detailed investigation of structure-function relationships still needs to be conducted.

## Effects of Sub-Inhibitory Concentrations of Antimicrobial Peptides at Cellular Level

When used in their non-lethal concentration, antimicrobial peptides have a powerful effect on the functioning of a bacterial genome, which ultimately leads to a change in the entire behavior of the bacterial population, provoking negative or positive effects for interrelated living organisms.

The bacterial envelope is the first protective structure on the pathway of antimicrobial peptides. AMP’s interaction with bacterial shells changes their surface architecture provoking undesirable effects. Thus, *Shigella flexneri* can use cationic proteins produced by neutrophils to increase self-adhesion and promote invasion inside epithelial cells (Eilers et al., 2010; Ni et al., 2015). LL-37 at sub-inhibitory concentration was proven to change *Streptococcus pyogenes* surface architecture, provoking the formation of extracellular vesicles, which contain numerous factors of streptococcal virulence (Uhlmann et al., 2016).

In Gram-positive bacteria, some virulence factors are assembled and attached to the cell wall by sortase enzymes, which are localized on one or two sides in the cell membrane. Several antimicrobial peptides can interact with focal sites and disrupt the localization of some proteins necessary for secretion and virulence factor assembly (Kandaswamy et al., 2013). For example, polymyxin B and HNP-1 at sub-MIC concentrations can bind to the anionic lipids of so-called ExPortal. It leads to structural disorder and effects cysteine protease and cytolysin secretion (Vega and Caparon, 2012).

The process of a microorganism’s conquest of a new habitat is accompanied by an appropriate reorganization of its metabolic processes. The presence of antimicrobial peptides at this point can either trigger the secretion of virulence factors that enhance the aggressiveness of the pathogenic microorganism, or decrease the metabolic activity and the appearance of persisters aimed surviving under the stress.

AMP-dependent sequential activation of PhoQP > PmrAB > ArnC leads to modification of lipid A (development of AMP-resistance) and at the same time, increased expression of the virulence factor PagC, necessary for bacterial persistence within macrophages (Yu and Guo, 2011; Tsai et al., 2016). The presence of LL-37 at sub-MIC led to the diversification of the *P. aeruginosa* population to the mucoid type, which increased their persistence and subsequently promoted chronic infection (Limoli et al., 2014). A similar result was revealed for *P. aeruginosa* population, growing in sputum of cystic fibrosis under sub-inhibitory concentrations of colistin (Wright et al., 2013). Another example of bacterial persistence is the induction of protective substances the function of which is inactivation of host defense antimicrobial proteins. For example, the human serum has numerous antimicrobial peptides and proteins, including lysozyme. The inhibition of lysozyme activity is one of the main causes of bacterial persistence (Bukharin et al., 1987). It was proven that the ability for induction of the main lysozyme inhibitor proteins Ivy and MliC is widespread in bacterial world and is under control of Rcs-regulon (Callewaert et al., 2009; Figure 1).

In addition, a good illustration of non-linearity and unpredictability of AMPs’ effects is the inhibition of toxin production in bacteria. *S. aureus* is one of the main pathogens of nosocomial infections, and methicillin-resistant strains are a serious problem in antimicrobial therapy. *S. aureus* is able to secrete a set of different virulence factors that allow it to colonize a different habitat. However, it has been observed that staphylococci growing on a blood-containing medium did not produce any toxins (Schlievert et al., 2007). It was hypothesized that human blood contains a factor that suppresses toxin-production. Today, it is known that animals’ blood is a source of various peptides including hemocidins, which are the cationic peptide fragments derived from hemoglobin (Mak et al., 2000; Arroume et al., 2008; Vasilchenko et al., 2016). Further studies of the antitoxic effects of hemoglobin showed the ability of globin chains to inhibit all known types of Agr-quorum sensing systems of *S. aureus*. Surprisingly, downregulation of *agr*-genes allows *S. aureus* to colonize nasal passages (Liu et al., 2013). It turned out that *S. aureus* cells reduce production of some Agr-regulated proteases to avoid generation of hemoglobin-derived antimicrobial peptides.

Finally, it is worth noting cases when the change in gene expression does not lead to the expected phenotypic changes. For example colicin M induces an envelope stress response of *E. coli* which upregulated numerous biofilm-associated genes. Nevertheless, the induction of neither biofilm formation nor of colonic acid production was observed (Kamenšek and Žgur-Bertok, 2013). Inducing the expression of virulence genes, did not cause any expected phenotypic changes indicating that several cellular targets were affected. So, colicin M induced the up-regulation of numerous biofilm-associated genes of *E. coli*. At the same time, it promoted the hydrolysis of lipid II, which limited its availability for exopolysaccharide biosynthesis, including colanic acid (Liu et al., 2013).

## Antimicrobial Peptides as Signaling Molecules

### Dual Function of Small Oligopeptides: Antimicrobial QS-Autoinductors

A shift in AMP’s function from antibiotic to signaling is one of the side-effects of diluting to sub-inhibitory concentrations. It is known that β-lactam antibiotics in sub-MIC have quorum-inducing activities, which triggers the synthesis of quorum sensing-dependent pathogenicity factors (Liu et al., 2013; Deryabin and Inchagova, 2017). However, the reverse scenario is also possible, when the autoinducer exhibits bactericidal properties (Qazi et al., 2006).

The quorum sensing-dependent process of regulation of gene expression usually takes place in four stages, one of which receives the signal molecule, which provide a possibility to interference between cognate and non-cognate autoinducers (Ji et al., 1997). It makes sense, since autoinducers work not only within a single population, but are also involved in interspecies signal transduction (Lowery et al., 2008).

Among the various existing autoinducers, within the framework of this review, the most interesting group are small autoinducing peptides molecules (AIP). The chemical structure of AIPs is diversified into several types, such as small oligopeptides and cyclic lactone/thiolactone peptides (Singh et al., 2016). Thus, cyclic oligopeptides often combine an antimicrobial and a signal activity (Prasad, 1995). Some Lactobacilli produce a variety of antimicrobial small dipeptides, which inhibit the viability of bacteria, fungi and viruses, while also suppressing the production of bacterial exotoxins (Kwak et al., 2017). In particular, the culture filtrate of *Lactobacillus* contained numerous dipeptides including cyclo (L-Phe-L-Pro) having antifungal activity (Kwak et al., 2014). The ability of such molecules to suppress exotoxin production is related to their interference with cognate QS-autoinducers. It was shown that cyclo (L-Phe-L-Pro) dipeptide suppress the production of staphylococcal exotoxins (TSST-1) by interfering with the agr QS-system (Li et al., 2011).

This class of substances is relatively poorly studied, and aggregated information concerning they biological activity can be found in remarkable reviews devoted to precisely these substances (Prasad, 1995).

### Dual Function of High-Molecular-Weight Peptides: Antimicrobial Pheromones

As for ribosomally synthesized antimicrobial peptides, considering their role in signal transduction, it is first of all worth considering bacteriocins. Many bacteriocins are synthesized in a quorum-dependent manner (Kleerebezem and Quadri, 2001; Quadri, 2002). It is also known that co-incubation of several different strains significantly enhances production of bacteriocins (Maldonado et al., 2004). Apparently, the induction of bacteriocin synthesis in a mixed culture is widespread in nature, however, the role of inducers is usually taken by proteins or peptides that do not themselves have antimicrobial properties (Chanos and Mygind, 2016).

Can bacteriocins affect production of defense peptides in other species? To date, several bacteriocins that combine both antimicrobial and signaling properties are known, since their own biosynthesis is a quorum-dependent bacteriocin (Kuipers et al., 1995; Kleerebezem et al., 2004). The most studied one in this respect is plantaricin A (Hauge et al., 1998). The mechanisms of plantaricin A’s function as a pheromone and antimicrobial are different. The pheromone action of plantaricin A is initiated by electrostatic interaction with membrane lipids. Subsequent events include the spatial arrangement of the plantaricin A molecule in the lipid/aqueous phase interface, which allows the N-terminal residues to engage in a chiral interaction with its histidine kinase receptor (Kristiansen et al., 2005). Bactericidal activity of plantaricin A is realized when plantaricin’s concentration is increasing, which leads to a rearrangement into a alpha-helical conformation and penetration of a bacterial cell wall (Di Cagno et al., 2010). Nevertheless, the main function of plantaricin A is signaling, because concentrations, which are exhibited required for antimicrobial action are not achieved in nature (Dicks et al., 2018).

As expected, the spectrum of processes which are activated by bacteriocin’ autoinducers includes only synthesis pathways. However, proteomic studies of bacteria co-incubated with bacteriocin (plantaricin A, nisin) revealed a change in the production of proteins and peptides, which are involved in increasing the adaptive capacity of the strain in a multi-species community (Calasso et al., 2013; Mukherjee and Ramesh, 2015) and overcome a bacteriocin-containing environment (Miyamoto et al., 2015).

**FIGURE 2:**
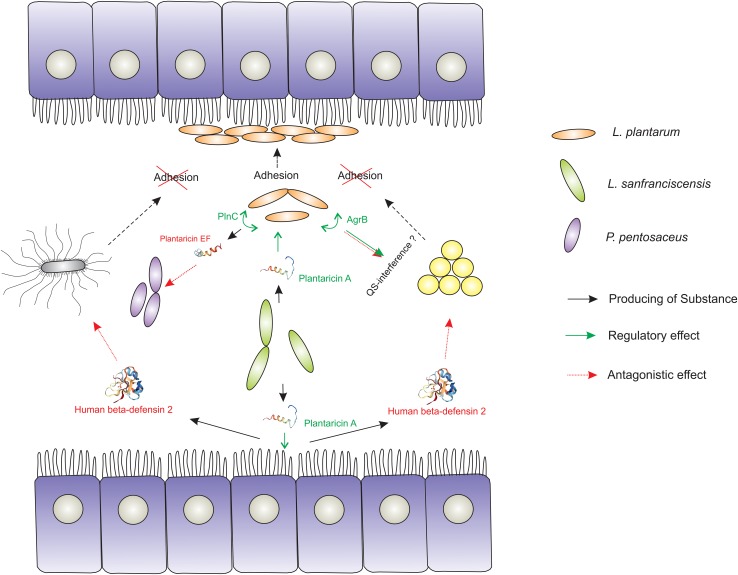
Demonstration of the signaling role of bacteriocins in the formation of a sustainable mammalian gut microbiome. The *Lactobacillus* strain producing plantaricin A triggers the expression of genes and the production of metabolites that enhance the colonizing ability (adhesion and biofilm formation) of another *Lactobacillus* strain. The occupied econish is no longer available for pathogenic and conditionally pathogenic microorganisms (*S. aureus* and *S. typhimurium*). In addition, certain (PlnC) activators of bacteriocin production (Plantaricin EF, for example) and some component of the agr QS-system (AgrB) are launched in susceptible to PlnA Lactobacilli cells, which has a certain antagonistic effect on the competitor species. Also plantaricin A triggers the production of human β-defensin 2 of the intestinal epithelium, which potentially has an antagonistic effect on a number of pathogenic and conditionally pathogenic microorganisms.

In addition, bacteriocin production stimulates the synthesis of human-defensin-2 (HBD-2) by the cells of the host intestine (Marzani et al., 2012), which also increases the colonization potential of certain species and provides ability for intra- and interspecies competition (Anderssen et al., 1998; Dicks et al., 2018; Figure 2). Thus, bacteriocins of one species can initiate the production of their own bacteriocins in another similar species. However, it seems that this induction of synthesis is caused by indirect action, since even insignificant structural differences between bacteriocins are critical for ligand/receptor interaction. Thus, subtilin does not interact with the histidine kinase NisK, which normally senses nisin, due to the differences between these bacteriocins in the structure of their N-terminal part (Spieß et al., 2015).

Describing the role of bacteriocins in microbial communities, it is necessary to mention the ability of bacteria to form biofilms. Biofilm is one of the characteristic forms of the existence of the multimicrobial community in nature (Sutherland, 2001). In nature, microbial cells exist in the attached state more often than in a free-floating planktonic state. Biofilms are structured by masses of microorganisms embedded in the matrix of polysaccharides, proteins, extracellular DNA and other molecules (Gillor, 2007). The development of bacterial biofilm is a quorum dependent phenomenon that ensures the viability of a bacterial population under adverse conditions.

It is known that bacteriocins have an important role in biofilm development. Bacteriocins inhibit the fixing of bacterial cells and the development of biofilms of competitive species when high local concentration is achieved (Gillor, 2007). At sub-inhibitory bacteriocin concentration a similar goal is also achieved, but in a slightly different way. For example, biofilm formation of *S. aureus* was abolished at sub-inhibitory concentrations of bovicin HC5 and nisin, because normal expression of genes associated with quorum sensing was affected (Pimentel-Filho Nde et al., 2014). Taken at sub-inhibitory concentration, subtilosin reduced biofilm formation of a conditionally pathogenic species *C. violaceum*. It was shown that subtilosin acts as a proton pump inhibitor in Gram-negative bacteria, which prevents efflux of a synthetized QS-autoinducer (Algburi et al., 2017). For more information about anti-biofilm properties of bacteriocins, the readers can be addressed to the recent review (Mathur et al., 2018).

There is an interesting point related to the fact that the action of bacteriocins, unlike most eukaryotic AMPs, is mediated through interaction with the corresponding receptors (Cotter, 2014). Numerous receptors, such as lipid II, are universal for a wide range of bacteriocins. In turn, certain molecules are receptors only for certain bacteriocins. Thus, lasso bacteriocin streptomonomicin interacts with WalR, a response regulator involved in cell wall metabolism and cell division (Acedo et al., 2018). Some thiopeptides interfere with protein synthesis either by binding to the 50S ribosomal subunit or elongation factors (Acedo et al., 2018). It is not yet clear what reactions can be triggered at the genome or secretome level when exposed to sub-inhibitory concentrations of such bacteriocins. Although it is known some antibiotics that inhibit protein biosynthesis in sub-inhibitory concentrations induce biofilm formation (Hoffman et al., 2005). There is also evidence that sub-inhibitory concentrations of glycopeptide vancomycin [cellular target is lipid II (De Moura et al., 2015)] change the expression of a several genes associated with virulence *E. faecalis* (Breukink and de Kruijff, 2006).

Thus, the main conclusions are:

1. Only cognate bacteriocins-pheromones can interact with appropriated receptors of regulatory systems.
2. The main function of such pheromones is the initial production of its own bacteriocins, and their antimicrobial properties is an additional feature.
3. However, it is possible that the range of biological effects initiated by bacteriocin-pheromones can be significantly wider than the production of its own bacteriocins (Xu et al., 2014). This presents a productive possibility for future research.

## Conclusion

In view of the above, the basic mechanisms for regulation of bacterial virulence factors have become more understandable. However, it is not yet possible to say exactly what happens with bacterial cells when sub-inhibitory doses of AMPs are exposed. Bacterial reaction on sub-MIC of AMPs can be non-linear. Yes, peptides are able to inhibit the production of any toxins, but it turns out that, subsequently, this ability is either restored, or one toxin is replaced by the production of another. Hemocidins reduce intracellular amounts of TSST-1, hemolysins, and lipase for *S. aureus* cells. However, the production of the virulence factor protein A is increased (Schlievert et al., 2007).

The presence of a multitude of sensory systems that are intertwined with each other allows bacteria to adapt to any stress. Thus, the reaction of bacterial pathogens to protective peptides consists of two parts: on one hand, the initial presence of a certain amount of AMP reduces the production of aggression factors and various exotoxins. On the other hand, a decrease in the microbe’s enzymatic activity provokes their persistence.

Throughout their evolutionary pathway bacteria have demonstrated a highly adaptive potential compared to other living organisms. In part, this has been the cause behind the current problem of antibiotic resistance, against which the efforts of many scientific groups are directed. Previously, it was believed that bacteria are significantly less resistant to the action of antimicrobial peptides than to conventional antibiotics, but today it is known to be not entirely true. Bacterial populations often respond to stressful effects unpredictably, and peptide action can both weaken the virulent potential of microbes as well as substantially increase it. The specific scenario will depend on the peptide’s properties and its local concentration. These factors are very poorly studied. For the realization of antimicrobial peptides’ potential as therapeutic agents, it is necessary to study their non-lethal effects on the physiology and behavior of microorganisms in the same way as the mechanisms of lethal action.

## Author Contributions

AV designed the review and wrote the first draft of the manuscript. ER reviewed and edited the manuscript. All authors contributed to manuscript revision and read and approved the submitted version.

## Conflict of Interest Statement

The authors declare that the research was conducted in the absence of any commercial or financial relationships that could be construed as a potential conflict of interest.

## Footnotes

1 www.pubmed.gov
